# INFLUENCE OF AGE ON PARAMETERS FOR FEMOROACETABULAR IMPINGEMENT AND HIP DYSPLASIA IN X-RAYS

**DOI:** 10.1590/1413-785220172505173951

**Published:** 2017

**Authors:** ULF-KRISTER HOFMANN, INGMAR IPACH, INA-CHRISTINE RONDAK, ROLAND SYHA, MARCO GÖTZE, FALK MITTAG

**Affiliations:** 1. Department of Orthopedic Surgery, University Hospital of Tübingen, Tübingen, Germany.; 2. Institute of Medical Statistics and Epidemiology, Technische Universität München, Munich, Germany.; 3. Department of Radiology, University Hospital of Tübingen, University of Tübingen, Germany, Tübingen, Germany.

**Keywords:** Hip dysplasia, Femoroacetabular impingement, Pelvis, Radiography, Osteoarthritis

## Abstract

**Objective:**

While several radiographic parameters have been established to describe the geometry and pathology of the hip, their reference values and clinical significance remain a matter of dispute. The present study tests the hypothesis that age has a relevant impact on radiographic hip parameters.

**Method:**

Pelvic antero-posterior views were measured for CE angle, Sharp’s angle, acetabular depth-to-width ratio, femoral head extrusion index, roof obliquity, caput-collum-diaphyseal (CCD) angle, and Murray’s femoral head ratio, and the values obtained were correlated with age.

**Results:**

Significant weak and moderate linear correlations (all Ps<0.001) were observed between age and CE angle (ρ=0.31), Sharp’s angle (ρ=-0.38), extrusion index (ρ=-0.22), CCD angle (ρ=-0.15), depth-to-width ratio (ρ=-0.38), and roof obliquity (ρ=-0.19), while Murray’s femoral head ratio (ρ=0.05; P=0.274) was not associated with age. Interestingly, the parameters describing the acetabulum all showed a relevant increase in coverage with age, leading to CE-angles well beyond 40° and a Sharp’s angle below 35° in a large portion of asymptomatic older adults.

**Conclusion:**

While a decrease in CCD angle with age is described in most orthopedic textbooks, the changes observed with age in acetabular geometry far exceed those measured at the femoral head-neck junction. We recommend considering these alterations that may be attributable to age when formulating a radiographic diagnosis. **Level of Evidence III, Diagnostic Studies - Investigating a Diagnostic Test.**

## RESUMO

**Objetivo:**

Embora tenham sido estabelecidos vários parâmetros radiográficos para descrever a geometria e a patologia do quadril, seus valores de referência e significância clínica continuam sendo uma questão controversa. O presente estudo testa a hipótese de que a idade tem impacto relevante sobre os parâmetros radiográficos do quadril.

**Método:**

As vistas pélvicas anteroposteriores foram medidas quanto ao ângulo CE, ângulo de Sharp, proporção entre profundidade e largura acetabular, índice de extrusão da cabeça do fêmur, obliquidade do lábio do acetábulo (teto), ângulo cabeça-colo-diafisário (CCD) e razão da cabeça do fêmur de Murray e os valores obtidos foram correlacionados com a idade.

**Resultados:**

Foram observadas correlações lineares significantes, fracas e moderadas (todos os P < 0,001) entre idade e ângulo CE (ρ = 0,31), ângulo de Sharp (ρ = -0,38), índice de extrusão da cabeça do fêmur (ρ = -0,22), ângulo CCD (ρ = -0,15), proporção entre profundidade e largura acetabular (ρ = -0,38) e obliquidade do lábio do acetábulo (ρ = -0,19), enquanto a razão da cabeça do fêmur de Murray (ρ = 0,05; P = 0,274) não foi associada à idade. Curiosamente, todos os parâmetros que descrevem o acetábulo mostraram um aumento relevante de cobertura com idade, levando a ângulos CE bem além de 40° e ângulos de Sharp abaixo de 35° em uma grande parcela de idosos assintomáticos.

**Conclusão:**

Embora a diminuição do ângulo CCD com a idade seja descrita na maioria dos livros didáticos de ortopedia, as alterações na geometria acetabular observadas com a idade ultrapassam, de longe, as medidas na junção entre cabeça e colo do fêmur. Recomendamos considerar essas alterações que podem ser atribuídas à idade ao formular um diagnóstico radiográfico. **Nível de Evidência III, Estudos Diagnósticos - Investigação de um Exame para Diagnóstico.**

## INTRODUCTION

Wiberg center-edge (CE) angle <25°, femoral head extrusion index >25%, Sharp’s angle >40°, acetabular roof obliquity angle >10°, and acetabular roof obliquity angle >10° have been established as a classic sign of hip dysplasia,^1^ ^,^ ^2^ a predominant prearthrotic deformity. In recent years, other changes in hip geometry have been added to describe pathological anatomy of the hip joint. Terms such as excessive overcoverage, acetabular retroversion, and abnormal head-neck junction (“pistol-grip deformity”) are now the focus of scientific interest. These anatomical changes can cause two forms of femoroacetabular impingement (FAI) leading to early hip pain and osteoarthritis (OA):^3^ ^-^ ^6^ increased acetabular coverage (“pincer impingement”) causes damage at the acetabular rim, and an enlarged femoral neck (“cam impingement”) destroys the antero-superior area of the acetabulum.^4^ ^,^ ^7^ After detailed physical examination, a diagnosis of hip dysplasia or FAI is largely based on appropriate imaging. Different radiographic parameters in pelvic antero-posterior views have been established to detect these pathologies. Some have become quite popular over the past few years, such as the “pistol-grip deformity,” which is quantified as Murray’s femoral head ratio.^8^ ^-^ ^10^

In these conditions, over time progressive degenerative changes lead to osteophytes, narrowing of the joint space, subchondral sclerosis, and deformity of the bone ends, which in turn have a negative impact on the different radiographic parameters themselves.^11^ When evaluating radiographic parameters, however, age also needs to be considered as a factor. The caput-collum-diaphyseal (CCD) angle, for example, is known to decrease significantly with increasing age.^12^ Moreover, aged cartilage usually shows non-progressive changes: decreased cellularity, reduced proteoglycan concentration, and reduced mechanical properties. The present study was performed to investigate the hypothesis that not only OA, but also age itself has an impact on different radiographic parameters used to describe hip dysplasia or FAI. 

## MATERIAL AND METHODS

**Figure 1:**
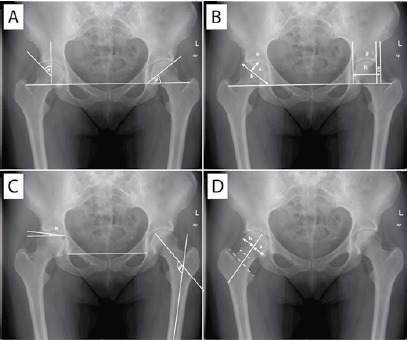
Measured radiographic parameters. ap = antero-posterior; L = left. A) *Wiberg's center-edge angle is defined as the angle between a line perpendicular to the horizontal teardrop line drawn through the center of the femoral head and a line from the center of the head to the lateral rim of the acetabulum. #Sharp's angle describes the angle formed by the horizontal teardrop line and a line from the inferior teardrop point to the lateral edge of the acetabulum. B) *The acetabular depth-to-width ratio is the ratio formed by the distance between the inferior teardrop point and the lateral acetabular rim (width) and the maximum perpendicular distance from this line to the acetabular wall (depth). #The femoral head extrusion index is the percentage of the femoral head that extrudes beyond the acetabular edge on a teardrop line plane (a:b). C) *The acetabular roof obliquity angle is formed by the line connecting the inferior-most edge of the roof of the acetabulum to the lateral-most edge of the acetabulum with a line parallel to the teardrop line. #The caput-collum-diaphyseal angle is measured between the longitudinal axes of the femoral shaft and neck. D) Murray's femoral head ratio is created by drawing a line through the middle of the femoral neck and the middle of the line connecting the apices of the greater and lesser trochanter. The perpendicular maximum distance from this line to the limit of the femoral head on each side is measured and the inferior distance divided by the superior distance (a:b).

We analyzed our data bank for all pelvic views performed in our institution between January 1, 2006 and December 31, 2011. The images were analyzed by a specialist registrar from our Department of Radiology. To avoid the negative influence of pelvic tilt and rotation on radiographic parameters, we included only standardized pelvic antero-posterior radiographs in the measurements. The mean distance between the tip of the coccyx and the middle of the symphysis was 32 mm for men and 47 mm for women, and the teardrop sign appeared to be symmetrical.^3^ To evaluate the severity of OA of the hip, we used the classification by Kellgren and Lawrence. Only radiographs with no signs of OA of the hip (Kellgren and Lawrence 0) were included in the present study. The pelvic antero-posterior views were measured for CE angle, Sharp’s angle, acetabular depth-to-width ratio, femoral head extrusion index, acetabular roof obliquity angle, CCD angle, and Murray’s femoral head ratio^8^ ^,^ ^10^ ^,^ ^13^ ^-^ ^17^ (for details concerning the measurement of these parameters, see Figure 1). Alpha-angle was not evaluated since recent studies have shown only a limited reliability for conventional radiographs and recommended it for 3D imaging techniques instead (reviewed by Sutter et al.^18^). In cases with unilateral total hip arthroplasty, fracture, or dysplasia (Crowe II-IV),^19^ only the contralateral side was measured. 

Full approval was obtained from the departmental, institutional, and ethical review boards (project number 025/2014R) before the study began. Due to the retrospective character of the study, no informed consent was obtained. 

### Statistical analysis

In order to account for repeated measurements of both hips on the patient level, we conducted analyses by using summarized values for both sides. Categorical variables are presented as frequencies and percentages, and continuous variables as means and standard deviations. The strength of linear associations between age and radiographic parameters was assessed by Pearson’s correlation coefficient (ρ). Linear or logistic regression analyses were conducted to describe the influence of age on the radiographic measurements. We considered the influence of possible confounding factors by calculating the corresponding odds ratio (OR) and 95% confidence interval (CI). Because of the descriptive character of the study, no alpha adjustment was performed with a two-sided significance level of 0.05. Statistical analyses were conducted using SPSS version 21 (IBM Corp., Armonk, NY) and R software, version 3.1.0 (R Foundation for Statistical Computing, Vienna). 

## RESULTS

Of all the pelvic radiographs performed in our institution between January 1, 2006 and December 31, 2011, those of 525 patients met all inclusion criteria. In 245 patients both sides were measured, and in 280 patients only the left (n=122) or right side (n=158) was measured, totaling a sample of 770 hip joints. The mean patient age was 50.6 (± 18.8) years; 48% of patients were male and 52% female. Protrusio acetabuli was detected in 4 hip joints, coxa profunda in 170 hip joints, and positive cross-over sign in 120 hip joints.

**Figure 2:**
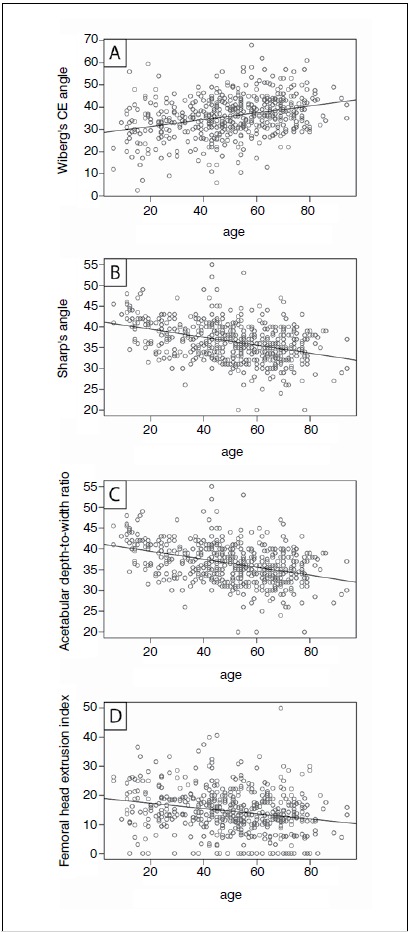
Pearson correlations between age and (A) Wiberg's center-edge (CE) angle, (B) Sharp's angle, (C) acetabular depth-to-width ratio, and (D) femoral head extrusion index.

**Figure 3:**
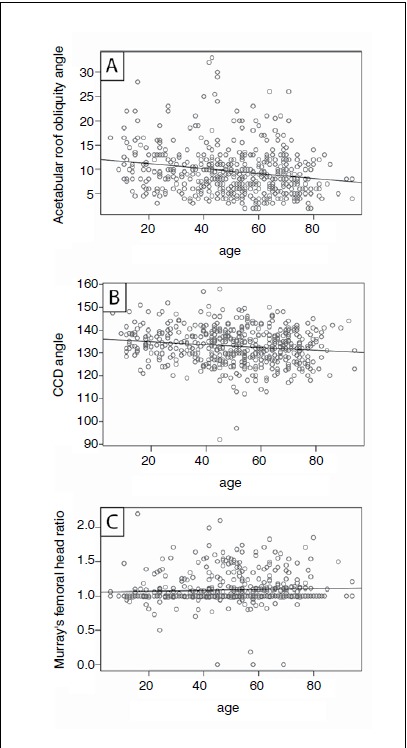
Pearson correlations between age and (A) acetabular roof obliquity angle, (B) caput-collum-diaphyseal (CCD) angle, and (C) Murray's femoral head ratio.

Significant weak or moderate linear associations (all Ps<0.001) were observed between age and CE angle (ρ=0.31), Sharp’s angle (ρ=-0.38), acetabular depth-to-width ratio (ρ=-0.38), femoral head extrusion index (ρ=-0.22), acetabular roof obliquity angle (ρ=-0.19), and CCD angle (ρ=-0.15). Murray’s femoral head ratio was not associated with age (ρ=0.05; P=0.274). (Table 1 and Figures 2 and 3) Linear regression analysis revealed a small negative effect of age on Sharp’s angle (β=-0.10), acetabular depth-to-width ratio (β=-0.10), femoral head extrusion index (β=-0.09), acetabular roof obliquity angle (β=-0.05) and CCD angle (β=-0.06), and a small positive effect on CE angle (β=0.15; each P<0.001) (Table 2). These results imply that age has a weak-to-moderate impact on the different radiographic parameters for FAI and hip dysplasia. No statistically significant influence of age on Murray’s femoral head ratio (β=0.00; P=0.274), protrusio acetabuli (left side: OR=1.03, 95% CI 0.95 to 1.12; right side: OR=1.04, 95% CI 0.97 to 1.14), coxa profunda (left and right sides: OR=0.99, 95% CI 0.98 to 1.01), or cross-over sign (left and right sides: OR=1.01, 95% CI 0.99 to 1.02) could be observed.

**Table 1:** Measurement values for the different radiographic parameters.

Hip parameter (n=770)	Mean (standard deviation)
Wiberg's CE angle	35.81° ± 9.56°
Sharp's angle	36.65° ± 4.79
Acetabular depth-to-width ratio	58.57% ± 7.94
Femoral head extrusion index	14.63% ± 8.04
Acetabular roof obliquity angle	9.49° ± 5.21°
CCD angle	133.36° ± 8.71°
Murray's femoral head ratio	1.09 ± 0.22

CE - center-edge; CCD - caput-collum-diaphyseal.

**Table 2:** Correlations and linear regression analyses of the effect of age on radiographic hip parameters.

Hip parameter (n=770)	Pearson correlation	Linear regression analysis
Wiberg's CE angle	ρ=0.31, P<0.001	β=0.15, P<0.001
Sharp's angle	ρ=-0.38, P<0.001	β=-0.10, P<0.001
Acetabular depth-to-width ratio	ρ=-0.38, P<0.001	β=-0.10, P<0.001
Femoral head extrusion index	ρ=-0.22, P<0.001	β=-0.09, P<0.001
Acetabular roof obliquity angle	ρ=-0.19, P<0.001	β=-0.05, P<0.001
CCD angle	ρ=-0.15, P<0.001	β=-0.06, P<0.001
Murray's femoral head ratio	ρ=0.05, P=0.050	β=0.00, P=0.274

CE - center-edge; CCD - caput-collum-diaphyseal.

## DISCUSSION

Hip dysplasia with reduced CE angle, decreased depth-to-width ratio, and increased extrusion index is widely accepted as the main reason for OA in young adults.^11^ ^,^ ^13^ ^,^ ^20^ Recently, other changes in acetabular geometry with excessive local or global overcoverage and reduced head-neck offset have been detected as further major causes for progressive hip pain and early OA of the hip. However, the predictability of these findings for early OA onset remains a matter of debate. On the one hand, abnormal hip morphology with either “classic” acetabular dysplasia or impingement due to excessive overcoverage of the femoral head, acetabular retroversion, or an abnormal head-neck junction has been reported in approximately 51%-97% of all cases of hip OA.^9^ ^,^ ^13^ On the other hand, Laborie et al.^9^ demonstrated a prevalence of radiographic findings for FAI in the majority of a cohort of 2081 healthy adults. De Bruin et al.^21^ found only 58 hip radiographs devoid of signs for FAI in a sample of 522 hips not clinically suspected of FAI. This leaves room to discuss the extent to which these parameters can be used as predictive factors for OA. Some of these findings may also be a consequence of early OA onset. For example, whether the posterior head tilt in osteoarthritic hips should be considered an acquired deformity created by the formation of osteophytes is currently under discussion. This has led to the suggestion that radiographic signs to detect FAI and hip dysplasia should be used carefully in patients with OA of the hip.^11^

The present study was performed to assess the impact of age itself on various radiographic parameters used to diagnose FAI and hip dysplasia. We showed that age has a significant influence on many of these measurements. With respect to the strength of the correlations with age, it is noteworthy that the decrease in CCD angle with age is described in most orthopedic textbooks. Nevertheless, except for the non-correlating Murray’s head-neck ratio, CCD angle showed the weakest correlation of all parameters analyzed (ρ=-0.15) in this study. It is noteworthy that while CCD angle and Murray’s head-neck ratio describe femoral changes, the parameters with much stronger correlation are all linked to the shape of the acetabulum. 

So far it is well known that Wiberg’s CE angle increases during skeletal growth. In adults, however, only a very weak increase has been described.^22^ The increase in acetabular coverage might seem accidental when just looking at one single parameter. The values observed are, however, all coherent. Just as Wiberg’s angle increases due to better coverage, Sharp’s angle, the acetabular roof obliquity angle, and the femoral head extrusion index decrease. And even though clinical experience might indicate that higher depth-to-width ratios are observed in older people (as in an osteoarthritic coxa profunda, for example), this conjecture is deceiving, since it only applies to osteoarthritic hip joints. With an increase in acetabular coverage, the resulting increase in width of the fossa exceeds the increase in depth, leading to a decrease of the ratio. How the radiological increase in acetabular coverage is produced still needs to be clarified. One possible explanation might be ossification of the labral base^23^ leading to false interpretation of the actual acetabular rim. It is more likely, however, that in the zone of maximum biomechanical stress the pelvis reacts over time by strengthening the apical zone. It is essential to realize that this acetabular increase leads to CE-angles well beyond 40° and a Sharp’s angle below 35° in a large portion of elderly people. These radiographic angles would usually be considered as FAI,^7^ ^,^ ^16^ but remain asymptomatic in many cases. Since the long-term outcome of surgical resurfacing of the head-neck junction still varies widely, we suggest considering age-related alterations before formulating a radiographic diagnosis from measured values. 

### Study limitations 

Some degenerative changes can almost always be observed in pelvic radiographs of older patients with hip pain. It is consequently difficult to make a clear distinction between whether these changes are attributable to ageing of the joint or to degeneration. In this study, however, only radiographs of patients not diagnosed with or treated for OA were included to minimize this effect. We did not test intra- or inter-observer reliability, although strong inter- and intra-observer discrepancies are known to occur in radiographic measurements to diagnose dysplasia and FAI. Even so, the lack of this evaluation should not have affected our results, since the only observer was blinded to patient age and considered all cases equally. Certain hip parameters may also be affected by a change in posture in elderly patients, but such a change in postural pelvic orientation would also affect joint function. Possible differences due to different projection angles in the radiographs may be solved in the future by using three-dimensional imaging techniques with volume renderings. 

## CONCLUSION

Patient age has a relevant impact on various radiographic parameters to detect FAI and hip dysplasia. While femoral CCD angle decreases only marginally, acetabular coverage increases considerably over time. Although these changes are in many cases negligible, especially when borderline values are found, alterations that may be attributable to age should be considered. 
